# It Would Cut into a Half Day

**Published:** 1916-04

**Authors:** 


### "It Would Cut Into a Half Day"

A while ago, a patient came with a history of obscure disease, dating back several years. After the preliminary examination, she objected to coming in office hours, for the above reason, although she was not a housewife and was, at the time, not working. A little further experience showed that her conception of medical attention was to run in at odd times, as convenient—to herself—to postpone or neglect appointments as fancy dictated and to observe no regularity of intervals between examinations. In fact, we have not yet succeeded in making a definite diagnosis although we have a fair idea of the reason of the chronicity of her ailment and of the prognosis.

Modern medicine is tending from symptomatic bedside treatment of disabled patients to the thorough analysis of chronic ambulant cases, formerly regarded as incurable even if amenable to relief. The average patient suffers less pain, is in less danger of life than formerly, but the solution of more recondite problems requires greater patience and more diligence on the part of both physician and patient. We must educate the laity to a conception of treatment as a serious piece of work, quite as important ultimately as any other, and requiring the same degree of regularity and promptness. It is worth while "to cut into a half day" for the sake of getting well.

